# Using Spectroradiometry to Measure Organic Carbon in Carbonate-Containing Soils

**DOI:** 10.3390/s24113591

**Published:** 2024-06-02

**Authors:** Piotr Bartmiński, Anna Siedliska, Marcin Siłuch

**Affiliations:** 1 Department of Geology, Soil Science and Geoinformation, Institute of Earth and Environmental Sciences, Maria Curie-Skłodowska University, al. Kraśnicka 2cd, 20-718 Lublin, Poland; marcin.siluch@mail.umcs.pl; 2 Institute of Agrophysics Polish Academy of Sciences, ul. Doświadczalna 4, 20-290 Lublin, Poland; a.siedliska@ipan.lublin.pl

**Keywords:** VIS-NIR spectrometry, carbonate soils, SOC

## Abstract

This study explores the feasibility of analyzing soil organic carbon (SOC) in carbonate-rich soils using visible near-infrared spectroscopy (VIS-NIR). Employing a combination of datasets, feature groups, variable selection methods, and regression models, 22 modeling pipelines were developed. Spectral data and spectral data combined with carbonate contents were used as datasets, while raw reflectance, first-derivative (FD) reflectance, and second-derivative (SD) reflectance constituted the feature groups. The variable selection methods included Spearman correlation, Variable Importance in Projection (VIP), and Random Frog (Rfrog), while Partial Least Squares Regression (PLSR), Random Forest Regression (RFR), and Support Vector Regression (SVR) were the regression models. The obtained results indicated that the FD preprocessing method combined with RF, results in the model that is sufficiently robust and stable to be applied to soils rich in calcium carbonate.

## 1. Introduction

Visible near-infrared spectroscopy (VIS-NIR) has become an increasingly widely used research tool in recent years [1,2,3]. The technique is based on the phenomenon of reflectivity, and the material under examination is exposed to electromagnetic radiation of a certain range and intensity. Specific chemical bonds absorb the radiation, reaching the object in different ways; by estimating how much radiation has been reflected from the object in the electromagnetic spectrum range from 400 to 2500 nm, the content of selected compounds can be determined indirectly. The visualization of the acquired information—a characteristic reflectance curve within which there are substance-specific peaks and valleys—provides a spectral signature that, when properly processed, can provide valuable qualitative and quantitative data. The acquired data require the necessary processing—preprocessing—for which various tools are used, such as curve smoothing, moving averages, etc. [4]. In many cases, the curves need to be corrected in specific ranges at the interface between the measurement ranges of the sensors used in the instrument; in a broad spectrum, it is impossible to use a single sensor. The data, prepared in an appropriate manner, are analyzed statistically, with the analysis usually studying either whole spectral curves or selected continuous fragments of them, dedicated—usually on the basis of previous scientific studies—to specific chemical compounds [5].

VIS-NIR spectroscopy is used in many fields: for instance, in agriculture, including precision agriculture, it is related to the appropriate application of the right amounts of substances necessary for plant growth and the assessment of the condition of the plant cover [6,7]. It is also used in geological studies, providing important information on, among other things, the mineralogical composition or the broader genesis of bedrock [8]. It can also be used indirectly to predict erosion risk [9].

A key feature of VIS-NIR spectroscopy is its high throughput, which allows a significant number of samples to be analyzed in a relatively short time. This feature plays a key role in projects requiring extensive research or the continuous monitoring of a given object [10]. The real-time analysis provided by VIS-NIR spectroscopy is another important advantage, enabling qualitative and quantitative assessments on site or in the field. This real-time feature is invaluable in situations where rapid or immediate decisions are required [11].

The quantitative analysis capabilities of VIS-NIR spectroscopy contribute significantly to the understanding of soil properties. The method can provide information on a range of parameters, including organic matter content [12,13], moisture levels [14] and nutrient concentrations [15], soil contamination with different types of pollutants [16], and electric conductivity [17]. Moreover, VIS-NIR spectroscopy is multifunctional and offers a holistic view by simultaneously assessing different soil characteristics, such as texture and mineral composition [18]. This comprehensive approach enables a detailed understanding of the conditions in a soil environment [19,20].

VIS-NIR spectrometric measurements are usually performed under controlled laboratory conditions. Samples are suitably prepared, above all in terms of material homogenization. The predictive models for individual soil properties obtained in the laboratory have a high accuracy. Attempts are made to carry out tests under field conditions, but the data obtained deviate significantly from the values obtained under laboratory conditions, and the predictive models developed are much weaker in this case [3,21]. Importantly, however, it should always be considered whether it is more important to obtain a better model or obtain information in an easier and much faster way.

In order to precisely identify characteristics, so-called spectral libraries are created [19,22]. Reflectance curves are collected for specific soils with specific characteristics, and these characteristics should include both the parameter directly covered by the spectral survey (e.g., organic carbon) as well as other soil characteristics that may have a significant impact on the spectral response (e.g., grain size, carbonates, mineralogical composition, etc.). A predictive model based on a broad spectral library should, in principle, produce higher-quality results. However, due to the enormous variability in soils on a global scale, the use of such libraries may be effective on a local scale or for specific soil types, under the additional assumption of limiting the availability of these studies.

Mapping spatial variability, facilitated by the integration of VIS-NIR spectroscopy with geospatial technologies, represents a higher level of soil survey methodology [23,24]. Creating detailed maps showing differences in soil properties within a given area is invaluable for precision agriculture and land use optimization. By identifying spatial patterns, this approach allows for targeted interventions, optimizing resource use and improving overall land management practices [25].

Among the soil parameters that have been determined by researchers is grain size, in varying aspects, both in terms of individual granulometric fractions and of the individual finest clay fraction [26], organic carbon content [27], soil color [28], salinity [29], and calcium carbonate content, sometimes presented as calcium carbonate equivalent [30,31].

Good results in terms of prediction based on spectral response have so far been achieved precisely for the analysis of soil organic carbon [32,33]. VIS-NIR spectrometry data can also be successfully used to determine the spatial variability in soil organic carbon saturation at the field scale [10]. Nevertheless, researchers indicate that the evaluation of the parameter in question can be affected by various other additional factors, such as aggregate structure, moisture content, content of specific substances, etc. [34,35]. One of the substances that could interfere and significantly affect the quality of SOC prediction is calcium carbonate; a significant interrelation of the two components in the samples studied, in terms of spectral properties, was pointed out by Rasooli et al. [36], among others. 

The purpose of this study is to determine the feasibility of analyzing soil organic carbon in carbonate-rich soils using VIS-NIR spectroscopy.

## 2. Materials and Methods

The materials for this analysis consisted of samples of soils, developed on the weathering of carbonate rocks—rendzinas—collected in Eastern Poland. The distinguishing feature of these soils is the high abundance of calcium carbonate and the associated high pH. All the soils at the time of collection were not in agricultural use and were perennial fallows, covered with vegetation of a natural-succession nature; nevertheless, no trees or shrubs were found. Soil samples were taken from the humus horizon of the soil, from the face wall of the excavated pit, from a depth of 5–15 cm, which ensured that the sample had not been disturbed due to processes on the ground surface, such as biological activity. The collected material was dried under room conditions and then sieved through a 2 mm sieve and placed in cardboard boxes. Chemical determinations were made on the material in the laboratory. Carbon was determined using a LECO TruSpec automatic analyzer, according to ISO 10694:1995 [37]. The analysis was performed in triplicate, in separate analytical samples. The carbonate (inorganic carbon) content was determined by volume, using a Scheibler apparatus, based on ISO 10693:2014 [38]. The proportion of organic carbon was at an average level, but the presence of carbonates affected the brightening of color, which, to some extent, masked the abundance of organic matter. The basic properties of the soils are summarized in Table 1.

Spectroradiometric data acquisition was carried out in laboratory conditions, using a Spectral Evolution RS-3500 instrument. The instrument’s operating range is between 350 and 2500 nm, and its resolution is 2.8nm (up to 700 nm), 8 nm (up to 1500 nm), and 6 nm (up to 2100 nm). The measurement was performed by contact, directly on samples homogenized immediately before the measurement, using a custom light source (Tungsten lamp). Due to the nature of the material tested (very light color), reflectance calibration was performed on a reference plate (Spectralon Reflectance Standard) before each measurement. At the end of the measurements, the results were digitally brought down to 1 nm resolution

### 2.1. Spectral Preprocessing

Prior to processing raw spectral data for the development of qualitative or quantitative analytical models in multivariate data analysis, mathematical transformations are applied. This essential preprocessing step is implemented to mitigate spectral variability and noise unrelated to the intended objectives of the models, while concurrently augmenting selectivity. In this study, two commonly used pretreatments for raw spectra, which included first (FD) and second (SD) using Savitzky–Golay smoothing filtering with 8 points and a polynomial order of 3, were selected. All the spectral pretreatments were performed in Python 3.8. To reduce the impact of low-intensity signals, two sections of the spectra spanning the complete wavelength spectrum were excluded: 350–400 nm. Thus, the spectral range from 400 to 2500 nm was retained for subsequent analysis.

### 2.2. Selection of Optimal Wavelengths

Due to the high dimensionality of hyperspectral data, selecting variables helps decrease the number of features to the most relevant ones. This process mitigates overfitting and enhances the predictive accuracy of regression models. For this investigation, Spearman correlation, Variable Importance in Projection (VIP), and Random Frog (Rfrog) were employed to select variables across three feature groups: raw reflectance, FD, and SD.

### 2.3. Spearman Correlation

A Spearman correlation analysis was conducted to evaluate the magnitude and direction of the monotonic relationship between the ranked response variable (the stem characteristics of each vine) and the ranked predictor variables (spectral data at various wavelengths). This analytical approach captures the tendency for paired variables to change in a synchronized manner, albeit not necessarily at a uniform rate, thereby facilitating the detection of nonlinear associations without imposing assumptions regarding the normal distribution of variables. The Spearman correlation coefficient, ranging from +1 to −1, signifies the strength of a monotonic relationship, with values closer to ±1 indicating stronger associations. Spearman correlation coefficients were calculated to indicate the relationships among SOC, SIN, raw reflectance, FD, and SD spectra. Variables exhibiting coefficients surpassing the threshold of 0.6 were deemed significant for inclusion in this study. The correlation analysis was conducted using “spearmanr” from the scipy library in Python 3.8.

### 2.4. Variable Importance in Projection (VIP)

The assessment of *VIP* is pivotal in multivariate analysis, particularly in the context of Partial Least Squares (PLS) modeling. The *VIP* selection method utilizes coefficients derived from a fitted PLS model to evaluate the significance of individual wavelengths (variables) within the dataset. In this method, key matrices including the X-score matrix (*T*), the y-loading vector (*q*), and the normalized X-weight matrix (W) are instrumental. Here, N represents the number of samples, *M* denotes the number of features, and K signifies the number of latent variables. The VIPs are computed using the following equation:

(1) $$VIP=\sqrt{M\frac{W^{2}{\left(q^{2}\;T^{t}\;T\right)}^{t}}{\sum_{k}{\left(q^{2}T^{t}T\right)}_{k}}}$$

This approach offers a comprehensive framework for assessing variable importance, aiding in feature selection and enhancing the interpretability of PLS models within scientific research and analysis [39]. Since the average of squared *VIP* scores equals 1, only influential wavelengths with a *VIP* score greater than 1 were kept in the calibration model.

### 2.5. Random Frog (Rfrog)

The Rfrog technique is an iterative selection method that commences with randomly chosen features, which are dynamically adjusted throughout the iteration process. During each iteration, a random subset or superset is generated and evaluated against the previously selected features through cross-validation. The Rfrog method maintains a counter for each feature, and the counters for all features within the “winning” set (i.e., achieving higher cross-validation scores) are incremented after each iteration. Following the completion of all iterations, the features with the highest selection frequencies are chosen for inclusion [40]. The number of iterations (N) was set to 50 in this study. In this study, VIP and Rfrog analyses were performed in Python 3.8 using the AUSWAHL (AUtomatic Selection of WAvelengtH Library) package.

### 2.6. Prediction Models

In this work, two types of datasets were proposed to achieve the best prediction accuracy for SOC estimation. The first type of dataset contained only spectral bands selected based on Spearman’s rank correlation coefficients and the VIP and Rfrog methods. In the second type of dataset, hyperspectral data were combined with information about the SIC obtained from laboratory measurements. As a result, sixteen different dataset combinations were utilized as inputs for the SOC prediction models, employing RF and PLSR algorithms.

### 2.7. Random Forest

RF is an ensemble regression technique that employs multiple decision trees. It constructs m decision trees from the training dataset using bootstrap resampling on m samples. Each decision tree split is built using a random subset of the dataset to measure a random subset of features in each partition [41]. This introduces variability among individual trees, thereby reducing the risk of overfitting and enhancing the overall prediction performance [42]. During the prediction phase, the algorithm aggregates the results of all trees by averaging, fostering a collaborative decision-making process supported by multiple trees and their insights. This approach yields stable and precise results, making Random Forests a versatile and reliable tool for various regression tasks. Throughout the training of the RF algorithm, a range of values were explored for the tuning parameters. Ultimately, the following parameter settings were selected: n_estimators = 10; max_depth = 20; and random_state = 101.

### 2.8. Partial Least Squares Regression Method (PLSR)

The PLSR algorithm amalgamates aspects of multiple linear regression analysis, canonical correlation analysis, and principal component analysis, offering not just a fitting regression model but also a comprehensive expression of information. The method operates under the assumption that the dependent variable can be estimated through a linear combination of explanatory variables [43]. Particularly advantageous in scenarios where numerous variables exhibit multiple correlations and the sample sizes are small, PLSR provides a many-to-many linear regression modeling approach. Unlike traditional classical regression analysis, which can lead to overfitting due to correlations among independent variables, PLSR identifies new linearly independent variables to replace the original ones, maximizing the difference between them.

The performance of the Rf and PLSR models was evaluated utilizing the Scikit-Learn python machine learning library package on the Windows (Spyder) platform.

### 2.9. Model Evaluation

In all dataset variants, the soil samples were divided at a 75:25 ratio into a calibration set and a validation set. For merged datasets containing hyperspectral and SIC data, the preprocessing phase involved standardizing the data to ensure compatibility with the analysis algorithms. Data integration plays a vital role when handling diverse data sources, often requiring merging and integration to create a cohesive and comprehensive dataset. To standardize the data, the Scikit-Learn library’s StandardScaler was utilized, providing a robust and efficient method for scaling features to a common mean and standard deviation.

The coefficient of determination (R^2^), the root mean squared error (RMSE), and the mean squared error (MAE) were calculated as indexes to evaluate the performance of the used models. Generally, a well-performing model tends to achieve a high R^2^ alongside low RMSE and MAE values, suggesting accurate predictions with minimal error.

## 3. Results and Discussion

The descriptive statistics of the soil organic matter and calcium carbonate equivalent in the soil samples are presented in Table 1 for both the calibration and validation sets. This includes calculations for the number of samples (N), the mean, the standard deviation (SDe), and the range. This finding indicated that the mean SOC for the calibration set and the validation set was 1.5% and 1.6%, respectively, whereas the average CaCO_3_ content in the calibration and validation set was 43.6% and 45.6%, respectively. The dataset distribution closely resembled that of the entire dataset, suggesting a representative division. Clearly, the inorganic carbon content in the form of calcium carbonate exceeded the organic carbon content by an order of magnitude. This is the distinguishing feature of carbonate soils in this type of rendzina.

**Figure 1:**
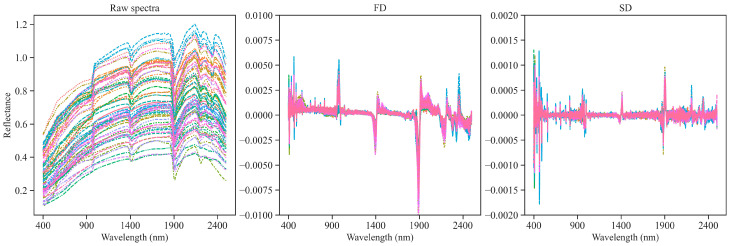
Raw and pretreated spectra. FD—first derivative; SD—second derivative.

Figure 1 shows raw spectra and pretreated spectra with FD and SD. In the raw spectra, consistent shapes can be observed across all the samples. The course of the curves is typical for soil material, as reported by many authors (LIT). Notably, three distinct absorption peaks are discernible in the near-infrared region, attributed to the hydroxyl group of free water (at 1410 nm and 1900 nm) and the Al-OH group of clay minerals, at 2210 nm [13]. 

Raw, unprocessed spectra showed reflectance in some sections exceeding the value of 1.0. This was due to the fact that the analyzed material had specific properties. First of all, the very bright color of the soil containing a high amount of carbonates resulted in a specific spectral response: the reflectance was locally higher than the reflection from the reference plate. In order to avoid error, calibration was performed before measuring each soil sample, as indicated in the Section 2. The highest recorded values, reaching 1.2 (reflectance at 120% relative to the reference), were recorded for soil samples containing up to 85% turbulent carbonates. This may be an important contribution to the discussion on the reference materials to be used for the heaviest soils (in terms of grain-size distribution) containing large amounts of inorganic carbon. In the literature, one does not encounter reflectance data exceeding 1. Nevertheless, the carbonate contents in the works analyzed are much lower (up to a maximum of 60%), and all the works showed very high correlations between reflectance and the amount of carbonate in the samples.

**Figure 2:**
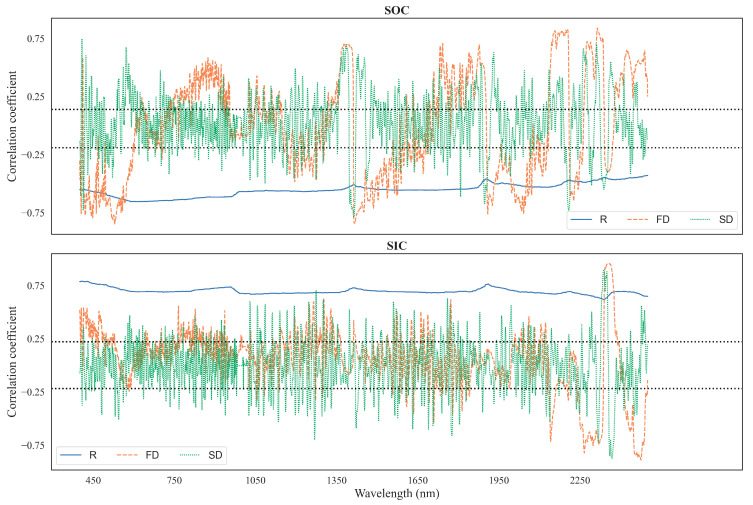
Spearman’s correlation coefficient distribution between the SOC and the SIC content with different preprocessing methods of spectral reflectance soil spectral data. The black dotted lines indicate significance at *p* < 0.01.

In Figure 2, Spearman’s rank correlation coefficients are presented for both the raw spectra and the spectral bands after FD and SD preprocessing. Notably, a significant negative correlation, approximately −0.7, was detected between the SOC content and the raw spectral data across the entirety of the spectrum range. Moreover, the SOC and the FD and SD spectra exhibited significant negative correlations within specific wavelength ranges, including 400–550 nm, 1400–1500 nm, and 1900–2000 nm. Conversely, a statistically significant and notably strong positive correlation was observed within the spectral regions spanning from 1700 to 1900 nm and from 2200 to 2500 nm. The strong correlation of the FD spectrum at 2300 nm was influenced by the characteristic absorption peak of C–H. However, a distinct response pattern was observed for SIC. Unlike SOC, a positive correlation was observed between the raw spectral data and SIC across the entire spectral range. A strong negative correlation was evident around 2300 nm and 2500 nm, while a strong positive correlation (higher than 0.75) was observed near 2400 nm. This indicated a contrasting relationship between the spectral data and the SIC compared to the SOC.

**Figure 3:**
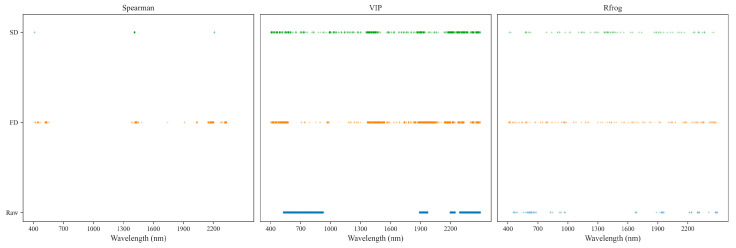
The distribution of spectral variables selected by a Spearman correlation analysis, VIP, and RFrog with different spectral pretreatments.

After conducting the Spearman correlation analysis, 187 wavelengths were identified for the FD spectra, while 19 wavelengths were deemed relevant for the SD spectra, all with a Spearman correlation rank higher than 0.6. These selected wavelengths are illustrated in Figure 3. For the FD spectra, the chosen wavelengths are aggregated into four ranges: 515–538 nm, 1420–1433 nm, 2165–2207 nm, and 2310–2333 nm. Meanwhile, the majority of wavelengths in the SD spectra are concentrated within the range of 1406–1417 nm.

**Figure 4:**
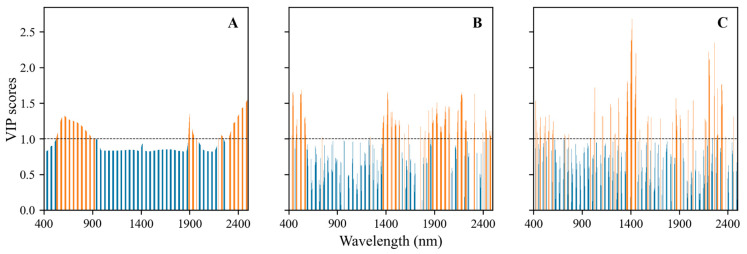
VIP scores of the wavelengths obtained for the raw (**A**), FD (**B**), and SD (**C**) spectra.

The VIP scores of the wavelengths obtained for the raw FD and SD spectra are depicted in Figure 4. In the case of the raw spectra (Figure 4A), three primary spectral zones were identified, spanning from 520 to 920 nm, around 1900 nm, and 2250–2500 nm. Additionally, five major spectral zones were distinguished as significant for the FD spectra, ranging from 450 to 570 nm, 1300–1520 nm, 1860–2600 nm, 2130–2200 nm, and 2270–2340 nm. Conversely, in the SD spectra, significance was observed across the entire spectrum. The implementation of the VIP method facilitated reduction, enabling the development of accurate and reliable models. The number of wavelengths decreased from 2100 to 765 for the raw spectra, resulting in a data size reduction of approximately 64%. Similarly, for the first- and second-order differentiations, the data size was reduced by approximately 57% and 61%, respectively. All the selected variables are illustrated in Figure 4. Four main spectral zones were observed for the raw spectra (525–927 nm, 1886–1974 nm, 2194–2250 nm, and 2287–2500 nm), while six spectral ranges were identified for the FD spectra pretreatments.

**Figure 5:**
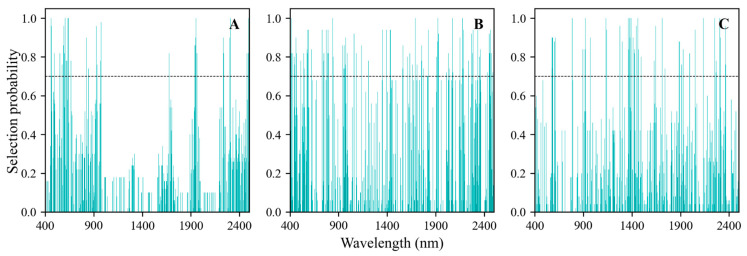
VIP scores of the wavelengths obtained for the raw (**A**), FD (**B**), and SD (**C**) spectra.

The feature wavelengths were selected by the Random Frog algorithm through the calculation of their selection probabilities within the spectrum. In Figure 5, the selection probabilities of each wavelength, determined by the Random Frog algorithm, are summarized for the raw and first- and second-order differentiations of the reflectance spectra. The threshold, established at 0.7 based on prior experience, was utilized to select important wavenumbers, with a selection probability surpassing this threshold as characteristic waves. Additionally, the number of model simulation iterations was set to 50 to ensure convergence. When employing a selection probability cutoff of 0.7, a total of 77, 169, and 93 significant wavelengths were identified for the raw, FD, and SD spectra, respectively (Figure 5).

With the hyperspectral data as an independent variable, two methods, Random Forest (RF) and Partial Least Squares Regression (PLSR), were employed to predict the SOC. The performance metrics of the proposed models utilizing various feature variable extraction methods are presented in Table 2. 

For all the studied variants, the prediction accuracies exceeded 65%. The models were constructed after band selection but still required fine-tuning to make better SOC predictions. The first derivative transformation of reflectance afforded the best predictions. The RF model attained the highest R^2^ value of 0.79 when employing variable extraction by Rfrog and preprocessing using FD. The model constructed based on the 19 wavelengths selected through Spearman’s correlation could predict the SOC with an R^2^ value of 0.77. 

The highest prediction accuracy was observed in the SOC prediction model based on the 169 wavelengths selected using the Rfrog method, with an R^2^ value of 0.79 and an RMSEP of 0.58%. Remarkably, the linear PLSR model demonstrated an inferior performance compared to the nonlinear RF model. The prediction models constructed using the PLSR algorithm demonstrated an adjusted validation R^2^ of between 0.41 and 0.75, with RMSE values of 0.98 and 0.64.

**Figure 6:**
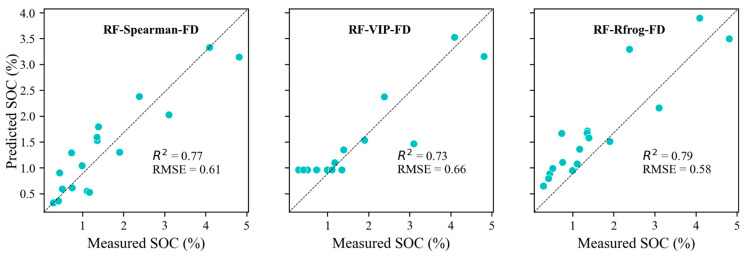
Scatter plots of the measured vs. predicted SOC values for different RF models based on spectral data. Black dotted lines represent the 1:1 lines.

Scatter plots depicting the predicted versus the measured values of SOC, generated by these high-quality models, are illustrated in Figure 6.

To enhance the predictive capability of the models, the hyperspectral data were integrated with the measured values of the SIC (Table 3). Compared to the model constructed solely based on the spectral data, the fused models showed an increase in their prediction accuracies of up to 20% and 13% for the RF and PLSR models, respectively. 

From Table 3, it can be noticed that the prediction accuracy, as indicated by R^2^, is satisfactory. However, there are notable discrepancies in the MAE and RMSE, attributed to the significant variability in the SOC samples. The best prediction model for the combined data was achieved with RF-Spearman-FD (R^2^ = 0.88; RMSE = 0.45). 

The obtained results indicated that the FD preprocessing method combined with RF, results in the model that is sufficiently robust and stable to be applied to soils rich in calcium carbonate. However, from Figure 2 and Figure 3, some discrepancy between the predicted and the measured values of the SOC content can be observed. The prediction of soil organic carbon (SOC) content is influenced by a range of environmental and management factors. Key factors affecting SOC prediction include the mineral composition and soil texture and, indirectly, soil structure, biological activity, vegetation cover, and climate conditions. These factors interact in complex ways, leading to spatial and temporal variability in the SOC content. Thus, effective SOC prediction models need to consider these diverse factors to improve accuracy and reliability. Due to the complex influence of many factors on the quality of organic carbon prediction, it should be taken into account that the laboratory testing of standardized soil samples (of a homogeneous structure, with water removed, free of plant debris and plant fragments) eliminates many factors that affect the results in unpredictable ways. Hence, testing in a systematic way, according to a specific protocol, allows one to achieve more reliable test results that are, in addition, directly comparable with the results of other researchers [44].

**Figure 7:**
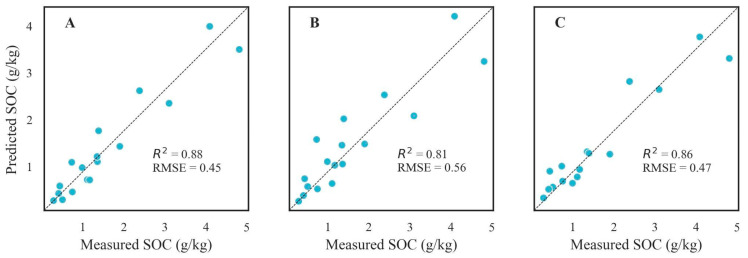
Scatter plots of the measured vs. predicted SOC values for different RF models based on combined datasets (spectral data and SIC). Black dotted lines represent the 1:1 lines.

Figure 6 and Figure 7 illustrate the precision of the prediction model, observable through the dispersion of points along the Y-axis. A narrower spread of these points around the predicted values signifies a higher precision. However, the observed scatter indicates that the model has certain limitations in its precision. This dispersion may stem from various factors, including inherent model constraints, data variability, and potentially unaccounted-for variables. Comparing prediction precision and instrumental measurement precision is vital for the validation and reliability assessment of the SOC prediction model. The precision of instrumental measurements, such as those obtained through the laboratory analysis of soil samples, serves as a benchmark for the prediction model. When instruments demonstrate a high precision, the SOC prediction model should ideally achieve a comparable precision to be considered reliable. Comparing the variability in the model’s predictions with the known precision of the instruments allows for a more detailed error analysis. If the model’s predictions show greater variability than the instrument’s measurements, this excess variability is likely due to the model’s limitations rather than issues with the SOC data’s quality.

Recognizing the precision of both the SOC prediction model and the instruments can inform targeted improvement strategies. For instance, if the instrument’s precision surpasses that of the model, efforts should focus on enhancing the model’s precision. This can be achieved by incorporating additional relevant features, refining existing algorithms, or exploring more sophisticated modeling techniques tailored to SOC data.

In summary, the precision of the prediction model, as depicted in Figure 6 and Figure 7, reveals certain limitations when compared with the higher precision typically associated with instrumental measurements. 

In summary, while prediction precision and instrumental measurement precision pertain to different domains, they share common principles, such as dependence on data quality, the necessity of validation and calibration, and the use of statistical methods to evaluate and enhance precision. Understanding their interconnection can lead to the better design and implementation of both predictive models and measurement systems, ensuring higher reliability and accuracy in various applications.

In light of the literature data, determining the precise wavelength at which a substance-specific signal is recorded can be debatable. For calcium carbonate, specific wavelengths have been determined: 1800 nm, 2350 nm, 2360 nm [45], 2325 nm [46], 2338 nm [47], 2340 nm [36], and 2341 nm (Gomez et al. 2008). However, in light of our analyses, the authors would suggest indicating wider ranges, related, on the one hand, to the diversity in soil samples and, on the other hand, the measurement method. The determination of a single length of the order of 1 nm, taking into account the spectral resolution of the apparatus at the level of 6 nm, seems methodologically unjustified. The reflectance/absorbance values at the suggested wavelengths may also be affected by the presence of certain clay minerals in the samples, such as chlorite or illite, which increase the absorbance of a given material in a similar range of the electromagnetic spectrum [47,48].

Organic carbon is one of the most commonly analyzed soil parameters using VIS-NIR spectrometry [7], considering the high importance of analyzing samples in the laboratory, with samples prepared in a specific way, which allows one to achieve more reliable results in contrast to analysis in the field. This is because in situ analysis must take into account local soil conditions, such as moisture content, structure, and, above all, the heterogeneity of the material [44]; the influence of the aforementioned factors is offset by the preparation of the material and its homogenization. The results of organic carbon prediction presented by many authors in available publications are highly promising and indicate the feasibility of using spectrometric techniques to analyze soil organic matter. R^2^ values, indicating the accuracy of prediction, at levels exceeding 0.8–0.9, should be considered satisfactory. 

Nevertheless, the selection of analytical material seems to be crucial in terms of prediction. Interfering factors are important, affecting the direct measurement of the spectral response of the soil in certain ranges or the specific “offset” of the entire spectral curve, due to high reflectance [36]. This is of great importance in the case of soils rich in calcium carbonate, such as those analyzed in this publication. The results obtained clearly indicate that it is necessary to use input fusion techniques, allowing researchers to take into account analytical laboratory results of calcium carbonate content. An increase in the quality of prediction using auxiliary data is also indicated in studies by other authors, who have taken into account, for example, soil moisture or temperature parameters [3].

One of the most important advantages of the VIS-NIR spectrometry method is that it facilitates and speeds up the analysis of soil materials. However, in the authors’ opinion, it is necessary, at least at this stage, to take into account traditional techniques (laboratory analytics) to improve the quality of prediction. In any case, adopting a compromise—analyzing calcium carbonate in the laboratory and incorporating the results into a combined prediction model—represents a cost-effective solution in terms of labor input and analytical costs. 

A methodological problem may be the relatively small database used for calibration, especially with a small total number of samples [49]. In the case of the present work, the number of samples used for calibration appeared to be sufficient [50]. On the other hand, increasing the measurement base would be difficult to achieve due to the uniqueness of the study material.

The comparison of the results obtained with those of other authors, in the case of VIS-NIR spectroscopy, often poses methodological problems. On the one hand, the analyzed material is highly diverse, including soils from different regions, with different basic properties (mineralogy, grain size, etc.). Nevertheless, it can be pointed out that most researchers obtain prediction results at an R^2^ level in the range 0.6–0.9 [7]. However, it seems that the published values cannot be generalized due to the high variability in the research material. The soils used in the present study, with carbonate contents exceeding 40%, have not yet been analyzed in detail in terms of organic carbon prediction by VIS-NIR spectroscopy. 

On the other hand, the processing of input data is performed in a differentiated way, which is due to the fact that authors are looking for the optimal solution (in the sense of the one producing the most reliable results). The use of diverse modeling techniques (e.g., Partial Least Squares Regression, cubist, Random Forest, Support Vector Machine, convolution neural network, XGBoost, memory-based learning, etc.) can lead to strongly divergent results in terms of prediction quality [4,51,52,53]. Consequently, there is no model solution that can be universally applied, but only a collection of individual case studies. Nevertheless, they provide an indispensable foundation for the creation of a library that takes into account different types and species of soils. The contribution of this publication in this regard is the inclusion of a particularly high carbonate content as an interfering factor in the organic carbon measurement range.

## 4. Conclusions

This study demonstrates the potential of VIS-NIR spectroscopy for SOC analysis in carbonate-rich soils. By integrating spectral data with SIC information and employing advanced modeling techniques, accurate predictions of SOC levels can be achieved, offering valuable insights for soil management and environmental monitoring.

In the case of some soils, it should be taken into account that, in certain ranges of the spectrum, reflectance may exceed the values for the reference materials.

## Figures and Tables

**Table 1:** Descriptive analysis of soil organic matter and calcium carbonate equivalent in soil samples.

Sample Set	n	SOC			CaCO_3_		
		Range [%]	Mean	Sd	Range [%]	Mean	Sd
All samples	68	0.1–5.1	1.5	1.2	0.1–86.1	44.1	25.4
Calibration set	51	0.1–5.1	1.5	1.2	0.1–86.1	43.6	26.4
Validation set	17	0.3–4.7	1.6	1.3	1.5–73.9	45.6	22.9

**Table 2:** The prediction results of SOC from the established RF and PLSR models, using Spearman, VIP, and Rfrog as data reduction methods on spectral input data.

Model	Feature Selection	Preprocessing	Calibration Set			Prediction Set		
			R^2^	MAE	RMSE	R^2^	MAE	RMSE
RF	–	-	0.88	0.28	0.42	0.51	0.59	0.89
		FD	0.96	0.18	0.25	0.79	0.43	0.59
		SD	0.91	0.27	0.37	0.82	0.42	0.54
	COR	FD	0.95	0.18	0.26	0.77	0.44	0.61
		SD	0.88	0.29	0.42	0.61	0.59	0.79
	VIP	-	0.91	0.26	0.36	0.38	0.69	0.99
		FD	0.90	0.28	0.38	0.74	0.51	0.65
		SD	0.92	0.25	0.34	0.59	0.60	0.81
	Rfrog	-	1	0.63	0.71	0.69	0.71	0.63
		FD	0.94	0.19	0.28	0.79	0.46	0.58
		SD	0.95	0.20	0.26	0.53	0.54	0.87
PLSR	–	-	0.27	0.74	1.02	0.41	0.69	0.98
		FD	0.55	0.61	0.8	0.65	0.61	0.76
		SD	0.57	0.57	0.79	0.62	0.79	0.57
	Spearman Cor	FD	0.64	0.72	0.53	0.73	0.50	0.66
		SD	0.51	0.56	0.74	0.66	0.56	0.75
	VIP	-	0.28	0.73	1.01	0.41	0.70	0.98
		FD	0.56	0.60	0.80	0.67	0.57	0.73
		SD	0.59	0.54	0.72	0.68	0.54	0.72
	RFrog	-	0.29	0.73	1.02	0.41	0.70	0.97
		FD	0.64	0.54	0.72	0.75	0.47	0.64
		SD	0.68	0.45	0.66	0.73	0.45	0.66

**Table 3:** The prediction results of SOC from the established RF and PLSR models, using Spearman, VIP, and Rfrog as data reduction methods on fused input data.

Model	Feature Selection	Preprocessing	Calibration Set			Prediction Set		
			R^2^	MAE	RMSE	R^2^	MAE	RMSE
RF	–	-	0.88	0.28	0.42	0.51	0.59	0.89
		FD	0.96	0.18	0.25	0.79	0.43	0.59
		SD	0.91	0.27	0.37	0.82	0.42	0.54
	COR	FD	0.95	0.27	0.18	0.88	0.32	0.45
		SD	0.93	0.32	0.23	0.77	0.46	0.61
	VIP	-	0.89	0.26	0.42	0.42	0.65	0.97
		FD	0.95	0.19	0.28	0.81	0.38	0.56
		SD	0.93	0.24	0.32	0.75	0.44	0.63
	Rfrog	-	0.87	0.28	0.44	0.54	0.61	0.86
		FD	0.95	0.20	0.28	0.86	0.32	0.47
		SD	0.93	0.24	0.32	0.58	0.62	0.82
PLSR	–	-	0.48	0.65	0.87	0.42	0.74	0.97
		FD	0.72	0.49	0.64	0.70	0.50	0.69
		SD	0.74	0.47	0.52	0.69	0.52	0.70
	Spearman Cor	FD	0.78	0.46	0.57	0.65	0.55	0.76
		SD	0.61	0.57	0.75	0.69	0.50	0.71
	VIP	-	0.53	0.65	0.83	0.51	0.60	0.89
		FD	0.78	0.45	0.56	0.77	0.45	0.61
		SD	0.80	0.42	0.54	0.74	0.51	0.65
	RFrog	-	0.56	0.63	0.79	0.55	0.57	0.85
		FD	0.81	0.40	0.52	0.81	0.40	0.55
		SD	0.84	0.37	0.48	0.68	0.61	0.72

## Data Availability

The data presented in this study are available upon request from the corresponding author.

## References

[1] Ben-Dor E. (2002). Quantitative Remote Sensing of Soil Properties. Advances in Agronomy.

[2] Ben-Dor E., Heller D., Chudnovsky A. (2008). A Novel Method of Classifying Soil Profiles in the Field Using Optical Means. Soil Sci. Soc. Am. J..

[3] Debaene G., Bartmiński P., Siłuch M. (2023). In Situ VIS-NIR Spectroscopy for a Basic and Rapid Soil Investigation. Sensors.

[4] Vestergaard R.-J., Vasava H.B., Aspinall D., Chen S., Gillespie A., Adamchuk V., Biswas A. (2021). Evaluation of Optimized Preprocessing and Modeling Algorithms for Prediction of Soil Properties Using Vis-nir Spectroscopy. Sensors.

[5] Santana F.B., Otani S., De Souza A., Poppi R. (2021). Comparison of PLS and SVM Models for Soil Organic Matter and Particle Size Using Vis-NIR Spectral Libraries. Geoderma Reg..

[6] García-Sánchez F., Galvez-Sola L., Martínez-Nicolás J.J., Muelas-Domingo R., Nieves M., García-Sánchez F., Galvez-Sola L., Martínez-Nicolás J.J., Muelas-Domingo R., Nieves M. (2017). Using Near-Infrared Spectroscopy in Agricultural Systems. Developments in Near-Infrared Spectroscopy.

[7] Ahmadi A., Emami M., Daccache A., He L. (2021). Soil Properties Prediction for Precision Agriculture Using Visible and Near-Infrared Spectroscopy: A Systematic Review and Meta-Analysis. Agronomy.

[8] Rosin N.A., Demattê J.A.M., Poppiel R.R., Silvero N.E.Q., Rodriguez-Albarracin H.S., Rosas J.T.F., Greschuk L.T., Bellinaso H., Minasny B., Gomez C. (2023). Mapping Brazilian Soil Mineralogy Using Proximal and Remote Sensing Data. Geoderma.

[9] Ostovari Y., Ghorbani-Dashtaki S., Bahrami H.-A., Abbasi M., Dematte J.A.M., Arthur E., Panagos P. (2018). Towards Prediction of Soil Erodibility, SOM and CaCO_3_ Using Laboratory Vis-NIR Spectra: A Case Study in a Semi-Arid Region of Iran. Geoderma.

[10] Reyes J., Ließ M. (2023). On-the-Go Vis-NIR Spectroscopy for Field-Scale Spatial-Temporal Monitoring of Soil Organic Carbon. Agriculture.

[11] Rodionov A., Welp G., Damerow L., Berg T., Amelung W., Pätzold S. (2015). Towards On-the-Go Field Assessment of Soil Organic Carbon Using Vis-NIR Diffuse Reflectance Spectroscopy: Developing and Testing a Novel Tractor-Driven Measuring Chamber. Soil Tillage Res..

[12] Nocita M., Kooistra L., Bachmann M., Müller A., Powell M., Weel S. (2011). Predictions of Soil Surface and Topsoil Organic Carbon Content through the Use of Laboratory and Field Spectroscopy in the Albany Thicket Biome of Eastern Cape Province of South Africa. Geoderma.

[13] Liu Y., Liu Y., Chen Y., Zhang Y., Shi T., Wang J., Hong Y., Fei T., Zhang Y. (2019). The Influence of Spectral Pretreatment on the Selection of Representative Calibration Samples for Soil Organic Matter Estimation Using Vis-NIR Reflectance Spectroscopy. Remote Sens..

[14] Liu J., Zhang D., Yang L., Ma Y., Cui T., He X., Du Z. (2022). Developing a Generalized Vis-NIR Prediction Model of Soil Moisture Content Using External Parameter Orthogonalization to Reduce the Effect of Soil Type. Geoderma.

[15] Cozzolino D., Cynkar W.U., Dambergs R.G., Shah N., Smith P. (2013). In Situ Measurement of Soil Chemical Composition by Near-Infrared Spectroscopy: A Tool Toward Sustainable Vineyard Management. Commun. Soil Sci. Plant Anal..

[16] Shi T., Wang J., Chen Y., Wu G. (2016). Improving the Prediction of Arsenic Contents in Agricultural Soils by Combining the Reflectance Spectroscopy of Soils and Rice Plants. Int. J. Appl. Earth Obs. Geoinf..

[17] Chatrenour M., Landi A., Bahrami H., Mirzaei S. (2023). Dust Source Clay Content and Salinity Estimation Using VNIR Spectrometry. Arid Land Res. Manag..

[18] Gholizadeh A., Žižala D., Saberioon M., Borůvka L. (2018). Soil Organic Carbon and Texture Retrieving and Mapping Using Proximal, Airborne and Sentinel-2 Spectral Imaging. Remote Sens. Environ..

[19] Viscarra Rossel R.A., Walvoort D.J.J., McBratney A.B., Janik L.J., Skjemstad J.O. (2006). Visible, near Infrared, Mid Infrared or Combined Diffuse Reflectance Spectroscopy for Simultaneous Assessment of Various Soil Properties. Geoderma.

[20] Debaene G., Bartmiński P., Niedźwiecki J., Miturski T. (2017). Visible and Near-Infrared Spectroscopy as a Tool for Soil Classification and Soil Profile Description. Pol. J. Soil Sci..

[21] Biney J.K.M., Borůvka L., Chapman Agyeman P., Němeček K., Klement A. (2020). Comparison of Field and Laboratory Wet Soil Spectra in the Vis-NIR Range for Soil Organic Carbon Prediction in the Absence of Laboratory Dry Measurements. Remote Sens..

[22] Ji W., Li S., Chen S., Shi Z., Viscarra Rossel R.A., Mouazen A.M. (2016). Prediction of Soil Attributes Using the Chinese Soil Spectral Library and Standardized Spectra Recorded at Field Conditions. Soil Tillage Res..

[23] Wetterlind J., Stenberg B., Söderström M. (2010). Increased Sample Point Density in Farm Soil Mapping by Local Calibration of Visible and near Infrared Prediction Models. Geoderma.

[24] Alomar S., Mireei S.A., Hemmat A., Masoumi A.A., Khademi H. (2022). Prediction and Variability Mapping of Some Physicochemical Characteristics of Calcareous Topsoil in an Arid Region Using Vis–SWNIR and NIR Spectroscopy. Sci. Rep..

[25] Feyziyev F., Babayev M., Priori S., L’Abate G. (2016). Using Visible-Near Infrared Spectroscopy to Predict Soil Properties of Mugan Plain, Azerbaijan. Open J. Soil Sci..

[26] Tümsavaş Z., Tekin Y., Ulusoy Y., Mouazen A. (2019). Prediction and Mapping of Soil Clay and Sand Contents Using Visible and Near-Infrared Spectroscopy. Biosyst. Eng..

[27] Hong Y. (2018). Application of Fractional-Order Derivative in the Quantitative Estimation of Soil Organic Matter Content through Visible and near-Infrared Spectroscopy. Geoderma.

[28] Zeng R., Rossiter D.G., Zhao Y.G., Li D.C., Zhang G.L. (2020). Forensic Soil Source Identification: Comparing Matching by Color, Vis-NIR Spectroscopy and Easily-Measured Physio-Chemical Properties. Forensic Sci. Int..

[29] Zhou M., Chen G., Dong Z., Xie B., Gu S., Shi P. (2020). Estimation of Surface Albedo from Meteorological Observations across China. Agric. For. Meteorol..

[30] Lagacherie P., Baret F., Feret J.-B., Madeira Netto J., Robbez-Masson J.M. (2008). Estimation of Soil Clay and Calcium Carbonate Using Laboratory, Field and Airborne Hyperspectral Measurements. Remote Sens. Environ..

[31] Melendez-Pastor I., Navarro-Pedreño J., Gómez I., Koch M. (2008). Identifying Optimal Spectral Bands to Assess Soil Properties with VNIR Radiometry in Semi-Arid Soils. Geoderma.

[32] Ladoni M., Bahrami H.A., Alavipanah S.K., Norouzi A.A. (2010). Estimating Soil Organic Carbon from Soil Reflectance: A Review. Precis. Agric..

[33] Angelopoulou T., Tziolas N., Balafoutis A., Zalidis G., Bochtis D. (2019). Remote Sensing Techniques for Soil Organic Carbon Estimation: A Review. Remote Sens..

[34] Wu C.-Y., Jacobson A.R., Laba M., Baveye P.C. (2009). Accounting for Surface Roughness Effects in the Near-Infrared Reflectance Sensing of Soils. Geoderma.

[35] Bellon-Maurel V., McBratney A. (2011). Near-Infrared (NIR) and Mid-Infrared (MIR) Spectroscopic Techniques for Assessing the Amount of Carbon Stock in Soils—Critical Review and Research Perspectives. Soil Biol. Biochem..

[36] Rasooli N., Farpoor M.H., Mahmoodabadi M., Esfandiarpour-Boroujeni I. (2023). Vis-NIR Spectroscopy as an Eco-Friendly Method for Monitoring Pedoenvironmental Variations and Pedological Assessments in Lut Watershed, Central Iran. Soil Tillage Res..

[37] (1995). Soil Quality—Determination of Organic and Total Carbon after Dry Combustion (Elementary Analysis).

[38] (2014). Soil Quality—Determination of Carbonate Content—Volumetric Method.

[39] Favilla S., Durante C., Vigni M.L., Cocchi M. (2013). Assessing Feature Relevance in NPLS Models by VIP. Chemom. Intell. Lab. Syst..

[40] Li H.-D., Xu Q.-S., Liang Y.-Z. (2012). Random Frog: An Efficient Reversible Jump Markov Chain Monte Carlo-like Approach for Variable Selection with Applications to Gene Selection and Disease Classification. Anal. Chim. Acta.

[41] Breiman L. (2001). Random Forests. Mach. Learn..

[42] Grimm R., Behrens T., Märker M., Elsenbeer H. (2008). Soil Organic Carbon Concentrations and Stocks on Barro Colorado Island—Digital Soil Mapping Using Random Forests Analysis. Geoderma.

[43] Shen L.-T., Zhang F.-M., Huang J., Li Y.-P. (2020). Climate characteristics of day and night precipitation during the growing season in Inner Mongolia from 1961 to 2018. Arid Zone Res..

[44] Piccini C., Metzger K., Debaene G., Stenberg B., Götzinger S., Borůvka L., Sandén T., Bragazza L., Liebisch F. (2024). In-Field Soil Spectroscopy in Vis–NIR Range for Fast and Reliable Soil Analysis: A Review. Eur. J. Soil Sci..

[45] Ben-Dor E., Banin A. (1990). Near-Infrared Reflectance Analysis of Carbonate Concentration in Soils. Appl. Spectrosc..

[46] Summers D., Lewis M., Ostendorf B., Chittleborough D. (2011). Visible Near-Infrared Reflectance Spectroscopy as a Predictive Indicator of Soil Properties. Ecol. Indic..

[47] Khayamim F., Wetterlind J., Khademi H., Robertson A.H.J., Cano A.F., Stenberg B. (2015). Using Visible and near Infrared Spectroscopy to Estimate Carbonates and Gypsum in Soils in Arid and Subhumid Regions of Isfahan, Iran. J. Near Infrared Spectrosc..

[48] Clark R.N., King T.V.V., Klejwa M., Swayze G.A., Vergo N. (1990). High Spectral Resolution Reflectance Spectroscopy of Minerals. J. Geophys. Res. Solid Earth.

[49] Chen S., Xu H., Xu D., Ji W., Li S., Yang M., Hu B., Zhou Y., Wang N., Arrouays D. (2021). Evaluating Validation Strategies on the Performance of Soil Property Prediction from Regional to Continental Spectral Data. Geoderma.

[50] Debaene G., Niedźwiecki J., Pecio A., Żurek A. (2014). Effect of the Number of Calibration Samples on the Prediction of Several Soil Properties at the Farm-Scale. Geoderma.

[51] Wang Z., Chen S., Lu R., Zhang X., Ma Y., Shi Z. (2024). Non-Linear Memory-Based Learning for Predicting Soil Properties Using a Regional Vis-NIR Spectral Library. Geoderma.

[52] Zhou Y., Chen S., Hu B., Ji W., Li S., Hong Y., Xu H., Wang N., Xue J., Zhang X. (2022). Global Soil Salinity Prediction by Open Soil Vis-NIR Spectral Library. Remote Sens..

[53] Guerrero C., Wetterlind J., Stenberg B., Mouazen A.M., Gabarrón-Galeote M.A., Ruiz-Sinoga J.D., Zornoza R., Viscarra Rossel R.A. (2016). Do We Really Need Large Spectral Libraries for Local Scale SOC Assessment with NIR Spectroscopy?. Soil Tillage Res..

